# Supplementary material for: A Pilot Study of Bioenergetic Marker Relationships in Gulf War Illness: Phosphocreatine Recovery vs. Citric Acid Cycle Intermediates
Source: Int J Environ Res Public Health. 2021 Feb 9;18(4):1635. doi: 10.3390/ijerph18041635 (PMC7914405; doi:10.3390/ijerph18041635)

Supplement I

Figure S1a. PCr-R vs CAC Intermediates in Controls

Succinate

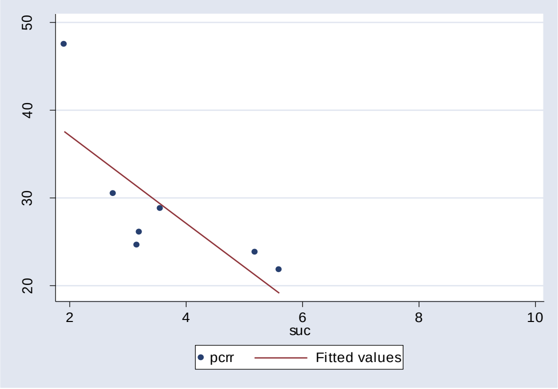

Malate

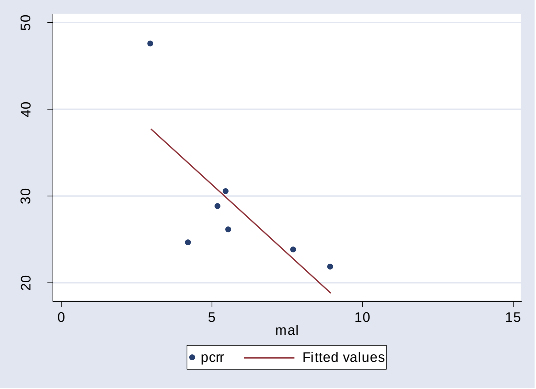

Fumarate

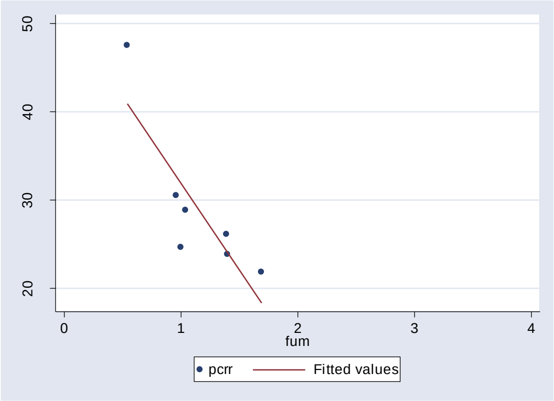

Citrate

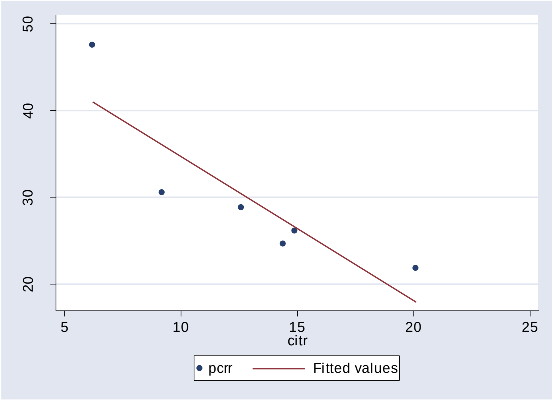

Isocitrate

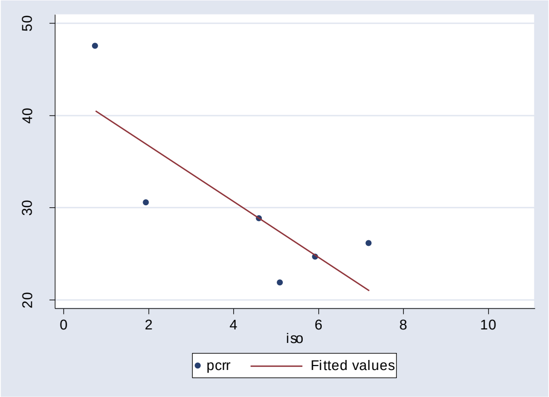

Alpha-ketoglutarate

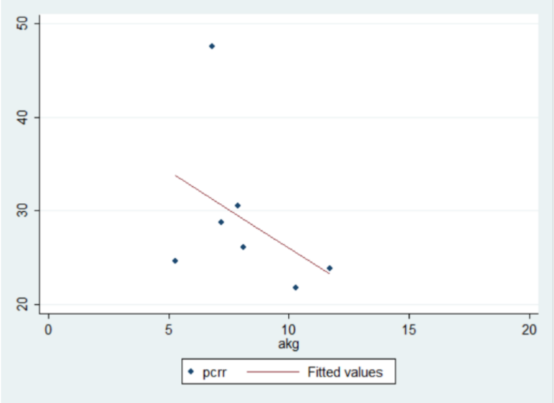

Aconitate

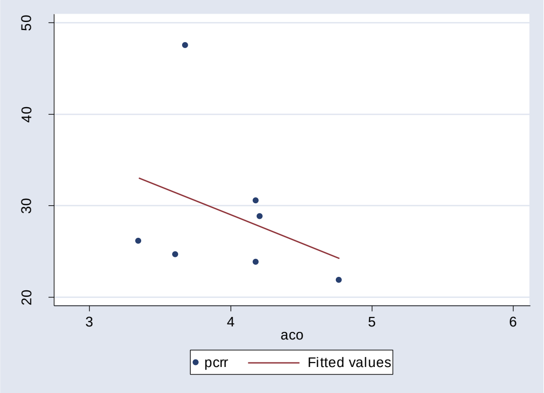

The only two CAC relationships to PCr-R in controls that were not significant, were for the two CAC intermediates (Alpha-ketoglutarate and Aconitate) that showed a relationship to archive time.

Figure S1b. Relations of PCr-R to CAC Markers in Cases. In cases, there is no apparent relationship

Succinate

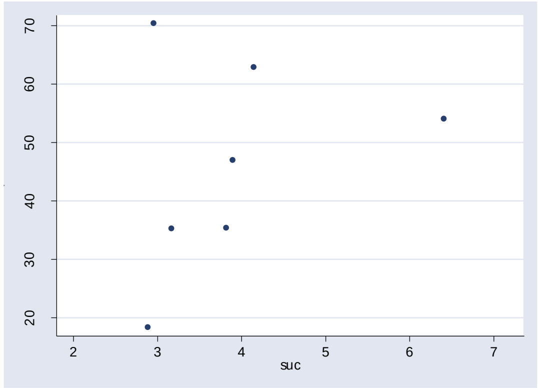

Malate

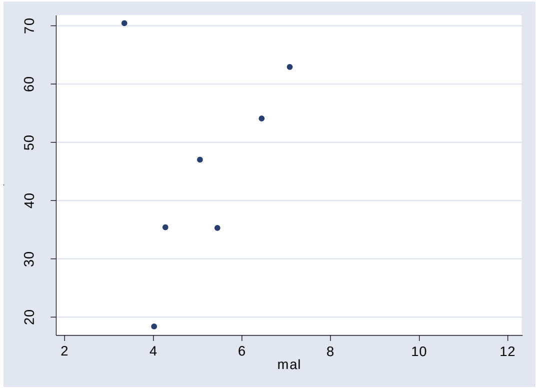

Fumarate

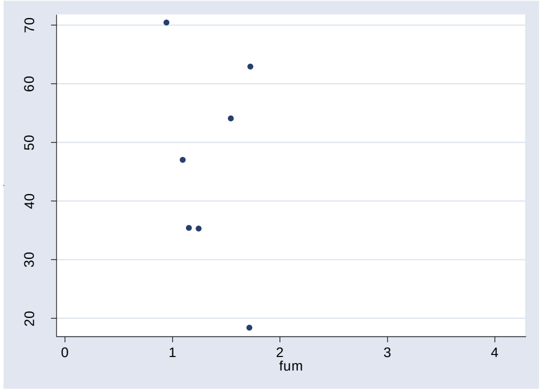

Citrate

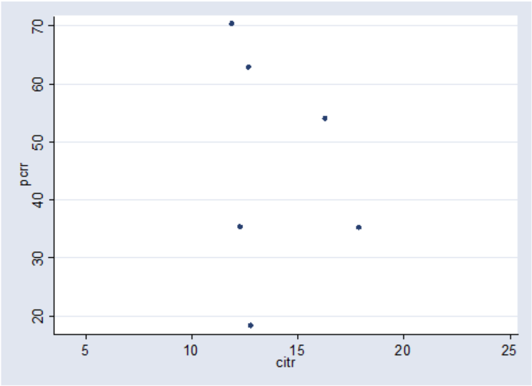

Isocitrate

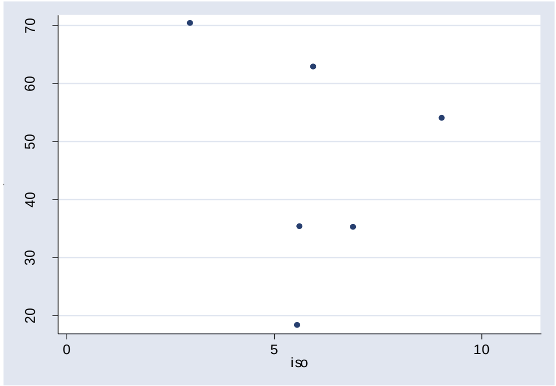

Alpha-ketoglutarate

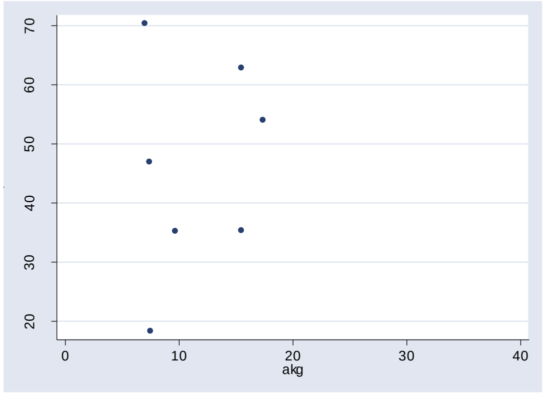

Aconitate

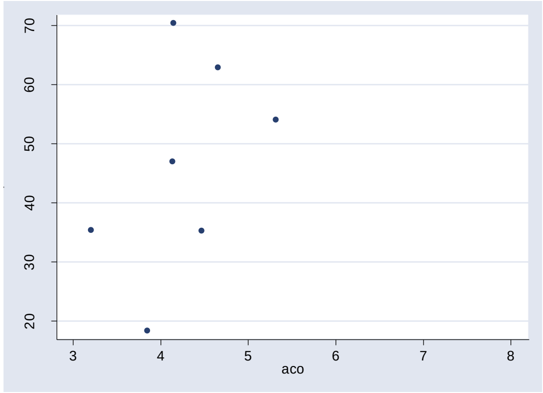

Supplement: Supplementary file 1 [file ijerph-18-01635-s001.pdf]
